# Effect of different mixing methods on the physical properties of Portland cement

**DOI:** 10.4317/jced.52893

**Published:** 2016-12-01

**Authors:** Shahriar Shahi, Negin Ghasemi, Saeed Rahimi, Hamidreza Yavari, Mohammad Samiei, Farnaz Jafari

**Affiliations:** 1 DDS, MSc, Professor, Dental and Periodontal Research Center, Dental Faculty, Tabriz University (Medical Sciences), Tabriz, Iran; 2 DDS, MSc, Assistant Professor, Department of Endodontics, Dental and Periodontal Research Center, Dental Faculty, Tabriz University (Medical Sciences), Tabriz, Iran; 3 DDS, MSc, Associate Professor, Department of Endodontics, Dental Faculty, Tabriz University (Medical Sciences), Tabriz, Iran; 4 DDS, MSc, Assistant Professor, Department of Endodontics, Dental Faculty, Tabriz University (Medical Sciences), Tabriz, Iran

## Abstract

**Background:**

The Portland cement is hydrophilic cement; as a result, the powder-to-liquid ratio affects the properties of the final mix. In addition, the mixing technique affects hydration. The aim of this study was to evaluate the effect of different mixing techniques (conventional, amalgamator and ultrasonic) on some selective physical properties of Portland cement.

**Material and Methods:**

The physical properties to be evaluated were determined using the ISO 6786:2001 specification. One hundred sixty two samples of Portland cement were prepared for three mixing techniques for each physical property (each 6 samples). Data were analyzed using descriptive statistics, one-way ANOVA and post hoc Tukey tests. Statistical significance was set at *P*<0.05.

**Results:**

The mixing technique had no significant effect on the compressive strength, film thickness and flow of Portland cement (*P*>0.05). Dimensional changes (shrinkage), solubility and pH increased significantly by amalgamator and ultrasonic mixing techniques (*P*<0.05). The ultrasonic technique significantly decreased working time, and the amalgamator and ultrasonic techniques significantly decreased the setting time (*P*<0.05).

**Conclusions:**

The mixing technique exerted no significant effect on the flow, film thickness and compressive strength of Portland cement samples.

** Key words:**Physical properties, Portland cement, mixing methods.

## Introduction

Portland cement is the main ingredient of Mineral Trioxide Aggregate (MTA). MTA is currently the most commonly used endodontic biomaterial, which is used for the repair of perforations, treatment of vital pulps and apexification (1-3).

Various studies have comprised the properties of Portland cement (PC) with MTA (4-9). The results have shown no differences in the antibacterial activities of these two materials (8). None of these two materials are cytotoxic or genotoxic and they both exhibit similar cellular reactions (10). The amount of arsenic released from both materials is similar and below the toxic levels (7). Inflammatory cells mount a similar reaction to both materials. The similar reaction of subcutaneous tissues of rates to these two materials indicates the similarity of the mechanism of action and the biologic reactions to these two materials, and histomorphological analyses have yielded similar results. The sealing ability of PC is similar to that of MTA (9). PC has been more successful in preventing microleakage than MTA (8) and has been successfully used for the repair of perforations and as a retrofilling material in a manner similar to MTA (9). A study reported that the marginal adaptation of PC was similar to that of White Mineral Trioxide Aggregate (WMTA). Both these materials are bioactive, which is attributed to the similar composition of these materials, except for the presence of bismuth oxide in MTA. In other words, the release of calcium hydroxide during the setting reaction of these two materials and the production of calcium phosphate is responsible for their biocompatibility (6).

These cements are hydrophilic; as a result, the powder-to-liquid ratio affects the properties of the final mix (11,12). In addition, the mixing technique affects hydration. Mechanical mixing in an amalgamator can decrease the number of air bubbles between the powder particles, resulting in complete wetting of the particles, which increases the homogeneity of the mix. Use of ultrasonic energy has attracted attention of researchers for mixing materials because such energy increases interaction of particles by extending the surface area of the particles participating in the setting reaction (13,14).

A study by Başturk *et al.* (11,12) showed increasing the compressive strength, hardness and density of MTA with the use of amalgamator and ultrasonic mixing techniques. Furthermore, the setting time of the final mix decreased by these methods. Shahi *et al.* (13-15) reported that the mixing technique had no effect on the film thickness, dimensional change, PH, push-out bond strength and compressive strength of the MTA; however, mixing with amalgamator increased the solubility and flow rate and also reduced the setting time and working time with the use of ultrasonic and amalgamator techniques.

Considering the favorable physical properties of PC, which are predominantly similar to those of MTA and also its low cost, many researchers believe that this material can be an alternative for MTA (6,8,9). The present study was undertaken to evaluate the effect of various mixing techniques on some selective physical properties (dimensional changes, film thickness, flow, setting time, working time, compressive strength, solubility and pH) of PC.

## Material and Methods

The design and method of this study were approved Tabriz Dental and Periodontal Research Center of the Tabriz University of Medical Sciences’ Investigation Committee (Grant no.65/6756).

-Preparation of samples

Before mixing the materials, the mixing pads, spatulas and the glass slabs were placed at 23±1°C temperature for 1 hour. The mixing procedures were carried out using conventional, amalgamator and ultrasonic techniques. In all the mixing methods the powder-to-liquid ratio was 3:1. In the ultrasonic technique, an ultrasonic scaling device (Juya Electronics, Iran) was used for mixing. In the amalgamator technique, proper ratios of the powder and liquid were transferred into the amalgamator container (Duomat II, Dental und Goldhalbzeug, 600 Frankfurt, Germany) and mixed for 20 seconds. For all the physical properties six samples were prepared for each mixing method (a total of 18 samples for each property). The methods used for evaluation of the physical properties were based on ISO 6876; 2001 and ISO 9917-1; 2003 specifications.

-Setting time

The materials were mixed and placed in cylindrical metallic molds, 15 mm in diameter and 5 mm in height. Within 2 minutes after initiation of mixing, the cylinders were placed in an incubator at a relative humidity of 100% at 37°C. After 30 seconds to 1 minute, the special plunger of the Vicat penetrometer (Schiller Park, Illinois, USA) was pressed perpendicular on the surface of each sample at a crosshead speed of 1 mm/min and maintained for 5 seconds. The procedure was repeated every 30 seconds until the plunger failed to produce a circular indentation on the sample surface. Setting time was defined as the duration of time from the beginning of mixing until the plunger failed to create a completely circular indentation on the material surface.

-Working time

Then 0.5 mL of the mixtures was placed at the center of a glass slab and then another slab was placed on it, with a 100-g weight placed on it for 10 minutes. Subsequently, the diameter of the sample was measured and the time during which the diameter reached a value 10% less than the original diameter was recorded as the working time.

-Dimensional changes

**Figure 1:**
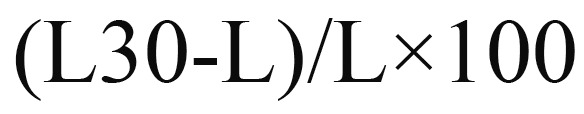
Formula

Each sample was placed in a Teflon mold measuring 12 mm in height and 6 mm in diameter and each mold was placed on a glass slab, measuring 75×25×1 mm, which was covered with a cellophane tape. The molds were a little overfilled and then a microscope plate covered with cellophane tape was placed on the top of each sample. This set was gently held by a piece of C-shaped pincers. Five minutes after initiation of mixing, the set was placed in an incubator under a relative humidity of 95% at 37°C for a duration 3 times longer than that recommended by the manufacturer for setting. Then both ends of each mold were abraded with wet 600-grit silicon carbide paper to create a flat and homogeneous surface. The samples were removed from the molds and a digital Vernier measuring tool, accurate to 0.01 mm, was used to measure their lengths. In the next stage, the samples were placed in a glass vial with 30 mL of deionized distilled water for 30 seconds at 95% relative humidity for 30 days. Then the samples were dried with absorbent paper and their lengths were measured again. The percentages of DC were determined using the following formula (Fig. 1).

in which L30 is the length of the sample after 30 days and L is the initial length of the sample.

-Film thickness

Under a relative humidity of 95% at 37°C, each sample was placed between two glass slabs after mixing using the technique described above. The overall thickness and surface area of the two glass slabs (5 mm and 200 mm2, respectively) were measured using a micrometer accurate to 1 μm. Three minutes after initiation of mixing, the glass slabs were placed in the IMI device (IMI Norgen Inc., Littleton, CO, USA) under a 150-N force in order to distribute the material evenly between the two slabs. After 10 minutes, the thickness of the whole set was measured using a micrometer. The test was repeated 3 times, with the mean being reported for each sample.

-Flow

Two mL of each mixed paste were placed at the center of a glass plate, measuring 40×40×5 mm 3 and weighing 20 g. After 3 minutes, another 100-g glass plate was placed on top of the material. The load was removed 10 minutes after the initiation of mixing, and the minimum and maximum diameters of the sample disks were measured using a digital caliper (Cole-Parmer Canada Inc, Montreal, Canada) accurate to 0.01 mm, followed by calculation of the mean values. In cases in which the disks were not uniformly circular (the maximum and minimum diameters were not within 1 mm), the test was repeated.

-Compressive strength

**Figure 2:**
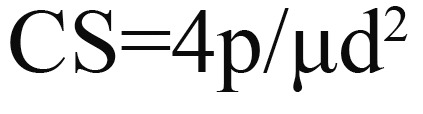
Formula

Sufficient amounts of the material were mixed using the three techniques and packed into steel molds, measuring 12 mm in height and 6 mm in diameter, within 2 minutes after initiation of mixing. Three minutes after mixing, the whole sets (molds and the samples) were placed in an incubator at 37±1°C and 100% relative humidity for 3 hours. Six samples from each mixing technique (a total of 18 samples) from each material, with no defects or bubbles, were selected for each time interval (21 hours and 21 days). The samples were stored in distilled water for 21 hours or 21 days and then underwent a CS test in a universal testing machine (Hounsfield Test Equipment, model: H5K-S, Perrywood Business Park, Honey Corckland, Salfords, Redhill, Surrey, UK) at a crosshead speed of 0.5 mm/min. The CS was calculated in MPa using the following formula, (Fig. 2).

where p is the maximum force applied in Newton, and d is the mean diameter of the specimen in mm.

-pH

A Metrohm 744 pH-meter (Metrohm Ltd, Herisa, Switzerland) was used to determine the pH values. The device was calibrated before the experiment using standard solutions with pH vales of 4 and 7. The mixed cements were placed in plastic tubes measuring 10 mm in length and 1.5 mm in diameter. The tubes were weighed accurately before and after being filled with cement samples. Each tube was separately placed in a flask with 10 mL of deionized distilled water for 1 hour at 37°C. Then an electrode was placed in the liquid-containing flask at 24°C and the pH value was registered.

-Solubility

**Figure 3:**
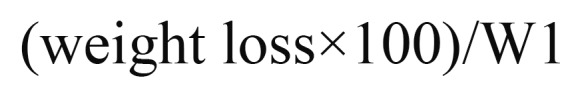
Formula

The mixed samples were placed within the disk-shaped molds measuring 20×1.5 mm. The samples were mixed and weighed by one operator at a relative humidity of 100%, followed by storage for 21 hours at 100% relative humidity. In the next stage, each sample was separately placed in glass bottles with 50 mL of distilled water at 37°C for 1 hour. Subsequently, all the samples were left to dry at 37°C for 1 hour and then weighed, after which the samples were returned to the same bottles with no changes in their water content. The drying and weighing steps of the samples were reported at 1-, 7- and 21-day intervals by subtracting W2 (the weight of the sample at the end of the related time interval) from W1 (the initial weight), indicating the weight loss. The amount of weight loss in μg was interpreted as solubility. The percentage of solubility was also calculated using the following formula, (Fig. 3).

-Statistical analysis

Data were analyzed using descriptive statistics (mean± standard deviation). Shapiro-Wilk test was used to evaluate normal distribution of data. Since the data was normally distributed, the one-way ANOVA was used to evaluate the significant effect of the material type and mixing methods. The post hoc Tukey’s test was used for the two-by-two comparison of the groups. SPSS software (SPSS version 22.0, SPSS, Chicago, IL, USA) was used for the analysis of data. The level of statistical significance was defined at 0.05.

## Results

 Table 1 presents the mean ± standard deviation (SD) of study data.

**Table 1:**
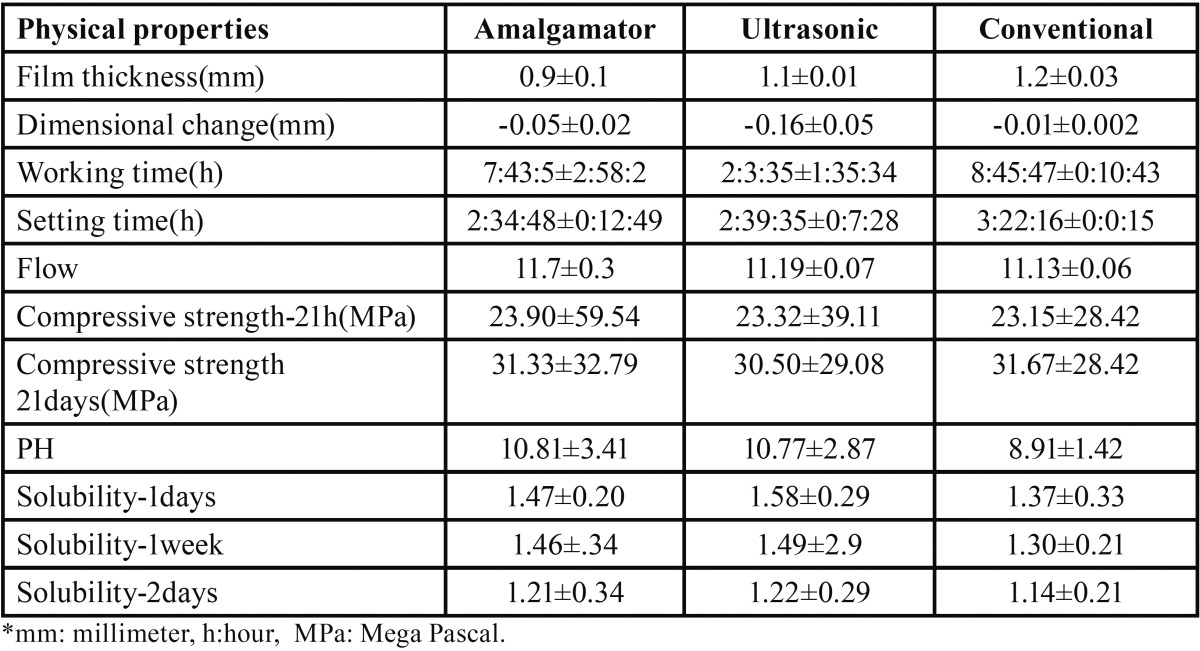
The mean ± SD of the data related to the study groups.

The mixing technique had no significant effect on the compressive strength, film thickness and flow of PC (*P*>0.05).

Dimensional changes (shrinkage), solubility and pH increased significantly by amalgamator and ultrasonic mixing techniques (*P*<0.05). The highest shrinkage, solubility and pH were obtained using ultrasonic mixing technique.

The ultrasonic technique significantly decreased working time, and the amalgamator and ultrasonic techniques significantly decreased the setting time (*P*<0.05). The conventional mixing method caused the highest amount of working time and setting time, significantly (*p*<0.05). Regarding the setting time there was no significant difference between the ultrasonic and amalgamator mixing techniques (*p*>0.05); but the difference between them was significant regarding the working time (*p*<0.05).

## Discussion

The aim of the present study was to evaluate the effect of different mixing techniques of the liquid and powder on some selective properties of PC. The results showed that the mixing technique, except for manual mixing, increased pH and shrinkage and decreased the setting time and working time; however, other physical properties were not affected.

An endodontic biomaterial should exhibit ideal physical properties for successful clinical application (12). To achieve these ideal properties in hydraulic cements like MTA and PC, the particles should be thoroughly mixed with liquid. In fact, the technique used to mix these materials results proper contact between the powder particles and the liquid; in other words, the mixing technique of these materials affects the hydration degree of the powder particles (15,16).

The solubility of calcium phosphate cements, and more specifically biomaterials such as mineral trioxide aggregate (MTA), has always attracted considerable attention (13). The ultimately important mechanism of osteo/dentino/cementogenic induction by PC is attributed to several factors, including its excellent sealing ability and alkalinity. On the other hand, the excellent biocompatibility of MTA is attributed to its alkaline pH and the potential to release calcium ions. Alkaline pH is important for hard tissue induction and antimicrobial activity considering that resistant endodontic bacteria, such as *Enterococcus faecalis*, are destroyed at a pH values over 11 (17). The results of our study showed that amalgamator and ultrasonic mixing techniques resulted in an increase in solubility and PH. Irrespective of mixing method, the overall pH of PC in this study is lower than the two other previous studies (6,18).

The compressive strength shows the progression of hydration process and is an indicator of the setting reaction of the material (12,19,20). The compressive strength is not considered important in cases in which a material is used as a retrofilling material; however, in cases of furcal perforation repair and apexification it is an important property because in such cases the material is affected by occlusal forces, and forces resulting from the placement of material are exerted on the material (11,14). This physical property depends on the liquid used for mixing, the pressure applied during placement of the material and the storage conditions of the material (12). In the present study, all the factors that affected the compressive strength were similar except for the mixing technique; and according to the findings, the strength of PC was not affected by the mixing technique, which is similar to the case with MTA (14). In comparison with the previous studies the value of compressive strength in our study is lower (4,6).

The setting time is one of the most important physical properties of biomaterials (15). The setting times of PC and MTA are one of their main disadvantages because they prevent one-visit treatments and contamination and washing away of the material is possible (21,22). In this investigation, the ultrasonic and amalgamator mixing techniques decreased the setting time of PC, which is similar to their effect on MTA. However, in none of the cases the decrease in the setting time is not adequate to make one-visit treatment possible. The mixing technique, the amount of liquid, the force used for packing and the environmental moisture affect the setting time (15); So, the samples were placed in the relevant cylinders by one operator and the storage conditions were the same for all the samples in our study. The obtained setting time is lower than values reported in similar studies (4,6,23).

Dimensional changes are under the influence of setting time, and a decrease in setting time results in a significant increase in dimensional changes (15). In the present study the dimensional changes in the PC appeared in the form of shrinkage with all the three mixing techniques. In the study by Islam *et al.* (6) the reported dimensional change of PC was expantion, in contrast with the present study. In our previous study on MTA, the dimensional changes appeared in the form of expansion (15); therefore, this property is different between these two materials. In fact, dimensional changes depend on soaking absorption and the water absorption properties of the material (6). When the dimensional changes of a material are in the form of shrinkage, there is a risk of loss of marginal adaptation, which might increase leakage. In the case of PC, there was less shrinkage in the manual mixing technique compared to the two other techniques, which should be taken into account when it is used as a retrofilling material.

Flow, film thickness and compressive strength were the other variables of the study, which were not affected by the mixing technique, which was similar to the results achieved with the MTA in our previous study. In this evaluation of the PC’s physical properties, the only variable that was negatively affected by the amalgamator and ultrasonic mixing techniques was the dimensional change that appeared in the form of shrinkage.
